# Effect of botanicals, organic nutrient sources, and bio-control agents on root-knot nematode (*Meloidogyne incognita*) infecting tomato

**DOI:** 10.3389/fpls.2025.1602326

**Published:** 2025-07-02

**Authors:** Vimala G., Mansi Machal, Virendra Singh Rana, Abhishek Gowda AP, Vijay Kumar, Najam Akhtar Shakil, Rashid Pervez, Ashish Kumar Singh, Ravinder Kumar, Mukesh Jaiman, Pankaj

**Affiliations:** ^1^ Division of Nematology, ICAR-Indian Agricultural Research Institute, New Delhi, India; ^2^ Division of Agricultural Chemicals, ICAR-Indian Agricultural Research Institute, New Delhi, India; ^3^ Division of Plant Pathology, ICAR-Indian Agricultural Research Institute, New Delhi, India

**Keywords:** root-knot nematode, Meloidogyne incognita, tomato (*Solanum lycopersicum*), biocontrol agents, green pesticides

## Abstract

Root-knot nematode (*Meloidogyne incognita*) causes up to 30% yield loss in tomato (*Solanum lycopersicum*) worldwide, and reliance on synthetic nematicides poses serious environmental and health risks. This study explores sustainable alternatives by evaluating the nematicidal potential of *Mentha* sp*icata* and *Piper longum* essential oils and extracts, along with *Bacillus amyloliquefaciens* and *B. subtilis*, under both *In vitro* and microplot conditions. Essential oils exhibited significant juvenile mortality and egg hatching inhibition at low concentrations, outperforming solvent extracts. In microplots, all treatments—including combinations with organic amendments (farm yard manure, vermicompost, and paddy straw)—significantly reduced nematode populations, improved tomato growth, and enhanced soil fertility. The combined biocontrol treatments performed comparably to the chemical nematicide Velum Prime 400 SC, while also increasing soil organic carbon and NPK content (P < 0.05). These findings demonstrate that integrating botanicals, plant growth promoting rhizobacteria and organic amendments provides an effective, eco-friendly alternative for managing root-knot nematodes, contributing to resilient and sustainable tomato production systems.

## Graphical Abstract

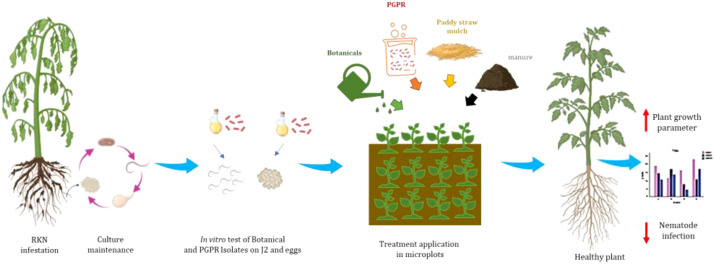

## Introduction

Tomato is the most widely commercially cultivated vegetable crop belongs to Solanaceae family. India ranks 2nd worldwide in both area and production of tomatoes, following China (Gupta et al., 2021). Tomato cultivation is hindered by several biotic and abiotic stresses, with root-knot nematodes (RKNs) as one of the most significant biotic threats. *Solanum lycopersicum* L. (Tomato), one of the most important crops is highly susceptible to root-knot nematodes, with infestations leading to yield losses of up to 30% (Sikora and Fernandez, 2005). In India the production of tomato suffers an estimated yield reduction of 23% translating annual financial loss of approximately ₹6,035.2 million due to RKNs suggesting the urgent need for sustainable and effective nematode management strategies (Kumar et al., 2020).

The management of root-knot nematode has traditionally relied on synthetic nematicides (Meher et al., 2010; Radwan et al., 2012; Pankaj et al., 2015). These nematicides effectively suppress nematode populations but their extensive use poses significant risks to human health, non-target organisms, and soil microbial diversity (Chen et al., 2020; Poveda et al., 2020; Sasanelli et al., 2021). In response, many countries have imposed stringent regulations on the use of synthetic nematicides (Jones, 2017), leading to a growing demand for sustainable, eco-friendly alternatives that ensure effective nematode control without compromising environmental and agricultural sustainability. “Green pesticides,” including botanicals, biocontrol agents, and organic amendments, present an eco-friendly and effective solution of nematode management.

Botanical extracts and essential oils have gained attention as eco-friendly alternatives for managing plant-parasitic nematodes due to their bioactive compounds with nematicidal properties (Saroj et al., 2020; Sarri et al., 2024). *Mentha* sp*icata* is well known for its natural insect-repelling abilities, effectively deterring and eliminating insect pests and mites through its essential oils and secondary metabolites (Mondal et al., 2024; Cai et al., 2025),. Similarly, the essential oil of *Piper longum* (long pepper) have been identified as safer alternatives for controlling of nematode and plant pathogens (Kumar et al., 2023; Páez and Leite, 2025), offering its nematicidal potential.

Biocontrol agents are preferred due to their specificity, environmental safety, cost-effectiveness, and non-resistance-inducing nature (Bhat et al., 2023; Chamkhi et al., 2022). It is reported that plant growth-promoting rhizobacteria (PGPR) suppress nematodes through multiple mechanisms, including competition, antibiosis, and induced systemic resistance in plants (Grewal et al., 2005; Pires et al., 2022; Antil et al., 2023). *Bacillus* species are widely studied and commercially utilized as biocontrol agents against plant-parasitic nematodes (Ramezani et al., 2014; Moreira et al., 2025; Ali et al., 2025). A combination of *Bacillus* species has shown to enhance nematode management more effectively than individual strains (Vasantha-Srinivasan et al., 2025).

The incorporation of organic nutrient sources, such as farmyard manure (El-Ashry, 2021), vermicompost (Gebrehana et al., 2025), and paddy straw (Wang et al., 2019), has been widely recognized for their role in enhancing soil fertility and improving plant resilience against nematode infestations (Khanna et al., 2022; Edussuriya et al., 2023).

A comprehensive review of the literature highlights the potential of eco-friendly alternatives such as botanicals, bio-control agents, and organic nutrient amendments in managing RKNs by improving soil health, enhancing plant resilience, and disrupting nematode life cycles. However, their individual efficacy in nematode suppression requires further validation. This study, therefore, evaluates the effectiveness of oils and extracts from *M.* sp*icata* and *P. longum* and two biocontrol agents, *Bacillus amyloliquefaciens* and *B. subtilis*, and organic amendments (farmyard manure, vermicompost, and paddy straw) against *M. incognita* in tomato. The findings provide valuable insights into sustainable nematode management strategies, offering environmentally safe alternatives to synthetic nematicides while supporting long-term agricultural productivity.

## Materials and methods

### Nematode culturing and inoculum preparation

Populations of *M. incognita* were collected from severely infected brinjal (*Solanum melongena*) plants maintained in microplots at the Division of Nematology, ICAR-IARI, New Delhi. The nematodes were identified based on perineal pattern, cultured and used to evaluate the bio-efficacy of organic nutrient sources and bio-control agents. Egg masses were carefully removed from infected roots using sterile forceps. The collected galls were placed in a modified Baermann funnel setup to facilitate nematode hatching. After 24 hours, freshly hatched second-stage juveniles (J_2_s) were collected in Petri plates containing sterile distilled water. To assess the effect of treatments on egg hatching, egg masses were extracted from infected roots and immersed in 0.1% sodium hypochlorite (NaOCl) for three minutes to release eggs. The suspension was then sieved through a 500 µm mesh screen. The eggs retained on the sieve were thoroughly washed with sterile distilled water to remove residual NaOCl (Hussey and Barker, 1973). The nematode suspension was transferred to a measuring cylinder, thoroughly mixed using a pipette, and concentrated to a specified volume to standardize the inoculum density. The total number of nematodes was estimated using a counting chamber, and the mean of three replicates was used for accuracy.

### Nematicidal activity of oils and extracts and biocontrol agents

In this study, different oils and extracts from *M.* sp*icata* and *P. longum* and plant growth-promoting rhizobacteria (PGPR) were evaluated for their efficacy against *Meloidogyne incognita* through *in vitro* and field conditions. The stock solution (1.0%) of the essential oils from the leaves of *M.* sp*icata* and fruits of *P. longum* and their hexane and methanolic extracts were prepared by dissolving them separately in distilled water and Tween-80 (3.0%) as surfactant. The solution was stirred continuously for 30 minutes to ensure homogeneity and was then diluted to obtain test of 2000, 1000, 500, 250, 125 and 62.5ppm. Distilled water with Tween-80 (3.0%) and Velum Prime were used as negative and positive controls. The prepared solutions were stored at room temperature (25 ± 2 °C) until further use.

### Preparation of biocontrol agents

The PGPR treatments consisted of *Bacillus amyloliquefaciens* DSBA 11, *B. subtilis* DTBS 5, and their combination and nutrient broth and sterile distilled water as controls. *B. amyloliquefaciens* DSBA-11 (ITCC BJ-0013) and *B. subtilis* DTBS-5 (ITCC BJ-0011) were obtained from the Bacteriology Lab, Indian Type Culture Collection (ITCC), Division of Plant Pathology, ICAR-IARI, New Delhi, India. A single colony from a 24-hour-old pure culture plate was inoculated into 50 mL of sterile King’s B broth in 100 mL Erlenmeyer flasks and incubated at 37°C with constant shaking at 150 RPM for 24 hours to promote bacterial growth. After incubation, the bacterial culture was centrifuged at 10,000 RPM for 15 minutes at 4°C to separate the supernatant. The cell-free culture filtrate was obtained by passing the supernatant through a 0.22 µm syringe filter and was subsequently used for bioassays after confirming the absence of viable bacterial cells.

### Egg hatching inhibition bioassay

The egg hatching inhibition bioassay was conducted using a 48-well culture plate, with each well containing *Meloidogyne incognita* egg suspension (100 eggs/10 µL). To each well, 1 mL solution of different concentration of oils and extracts (1000, 500, 325, 250, 125, and 62.5 ppm) and biocontrol agents (100%, 50%, and 25%) were added. The plates were incubated at 28 ± 2°C. Velum Prime^®^ (Fluopyram 400 SC) was used as the positive control, while Tween-80 (3.0%), nutrient broth, and sterile distilled water served as negative controls. Egg hatching was monitored at 2, 4, 6, and 8 days post-treatment using a stereoscopic binocular microscope. The percentage of egg hatching was calculated based on the ratio of hatched to unhatched eggs. Hatching inhibition was determined by comparing the number of unhatched eggs in treated samples to the control groups.

### Juvenile mortality bioassay

A 48-well culture plate was prepared by adding *M. incognita* suspension (100 second-stage juveniles (J_2_s)/10 µL) to each well. Subsequently, 1 mL of different concentrations of oils and extracts (1000, 500, 325, 250, 125 and 62.5 ppm) and biocontrol agents (100%, 50%, and 25%) were added, thoroughly mixed, and incubated at 28 ± 2°C. Velum Prime^®^ (Fluopyram 400 SC) was used as the positive control, while Tween-80 (3.0%), nutrient broth, and sterile distilled water served as negative controls. Nematode mortality was assessed at 24, 48, 72, and 96 hours post-treatment, with three replicates per treatment. The percentage of juvenile mortality was calculated based on the ratio of dead juveniles to the total juveniles in each well, following Abbott’s formula (Culver et al., 1925).

### Soil sample analysis for NPK and organic carbon

Soil samples were collected from micro plots at the Division of Nematology, ICAR-IARI, New Delhi, using a hand hoe, ensuring minimal disturbance to the soil structure. The collected samples were air-dried at room temperature, cleared of debris and roots, and sieved through a 2 mm mesh for uniformity. The processed samples were stored in labeled containers until further analysis. Soil nitrogen content was determined using the Kjeldahl method, which involved digestion, distillation, and titration (Bremner, 1960). Available phosphorus was extracted using the Olsen method and quantified calorimetrically using a spectrophotometer (Sims, 2000). Soil potassium was extracted with ammonium acetate and measured using flame photometry (Jackson, 1958). Soil organic carbon content was estimated using the Walkley-Black chromic acid wet oxidation method. All analyses were performed in triplicate to ensure accuracy and reproducibility. Standard reference materials and blanks were incorporated for instrument calibration and validation of results.

### Application of botanicals and PGPR as soil drenching

Bio-control agent isolates were applied to the soil by drenching at a rate of 100 mL/plot or 500 g/plot (10^8^ CFU/mL) two weeks before transplanting. After this period, 21-day-old Pusa Ruby tomato seedlings were carefully uprooted from nursery pro-trays and transplanted into each plot at a density of five seedlings per plot. Each treatment was replicated three times. Velum Prime^®^ (500 g a.i./ha) served as the positive control, while untreated soil was used as the negative control. Observations were recorded at 30, 60, and 90 days post-transplantation. At the end of the experiment (90 days), plants were carefully uprooted to assess growth parameters, including shoot length (cm), root length (cm), fresh shoot weight (g), and fresh root weight (g). Nematode infestation was evaluated by recording the number of galls per root system, number of egg masses per root system, and number of eggs per egg mass. Additionally, soil nutrient status, including nitrogen (N), phosphorus (P), potassium (K), and organic carbon content, was analyzed to assess the impact of treatments on soil fertility. The other treatment details for *in vivo* experiment under micro plot have been given in **Table 1**. Formulations of *B. amyloliquefaciens* and *B. subtilis* in both talc- and liquid-based forms were used either individually or in combination. These bioagents were applied after enrichment in well-decomposed farmyard manure (FYM). For enrichment, each bioagent formulation was thoroughly mixed with FYM and incubated for 15 days under moist conditions to promote microbial multiplication. The enriched FYM was then applied to the soil as per the treatment schedule.

**Table 1:** Treatment details for *in vivo* micro plot experiments.

Treatment Category	Treatment Code	Treatment Description
Organic Nutrient Sources	T1	Farm Yard Manure (FYM)
	T2	Vermicompost
	T3	Paddy Straw Mulch
Botanicals	T4	*Mentha* sp*icata* oil
	T5	*M.* sp*icata* methanolic extract
	T6	*Piper longum* hexane extract
	T7	*P. longum* methanolic extract
	T8	Velum Prime^®^ (500 g a.i./ha) as positive control
Plant Growth-Promoting Rhizobacteria (PGPR)	Ba-Talc	*B. amyloliquefaciens* (Talc) enriched in FYM
	Bs-Talc	*B. subtilis* (Talc) enriched in FYM
	Bs-Liquid	*B. subtilis* (Liquid) enriched in
	Ba-Liquid	*B. amyloliquefaciens* (Liquid) enriched in
	Ba + Bs (Liquid) + FYM	combination of *B. subtilis* (Liquid) + *B. amyloliquefaciens* (Liquid) enriched in FYM
	VP	Velum Prime^®^ (500 g a.i./ha) as positive control

### Experimental design and statistical analysis

Both *in vitro* and *in vivo* experiments were conducted using a completely randomized block design (CRBD). The recorded data were subjected to statistical analysis using the Web Agri Stat Package (WASP) version 2.0 at a 5% level of significance. The median lethal concentration (LC_50_) was determined using Probit analysis in the SPSS statistical package (version 16.0).

## Results

### Nematicidal activity of biocontrol agents

**Figure 1:**
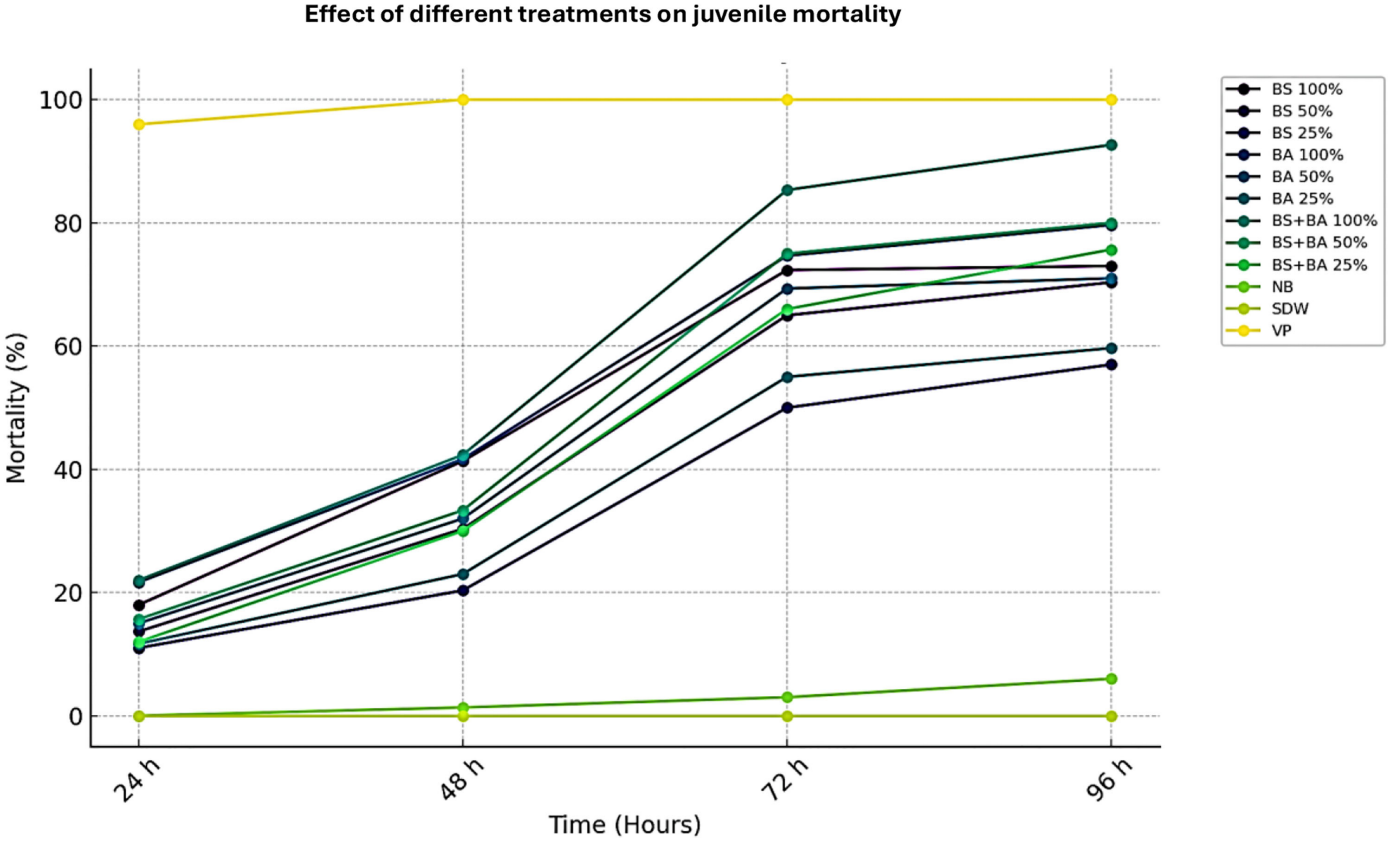
Bio-efficacy of different bio-control agents on juvenile (J2) mortality of *Meloidogyne incognita*. Abbreviations and Treatment Details: Ba, *Bacillus amyloliquefaciens* (strain DSBA 11); Bs, *Bacillus subtilis* (strain DTBS 5) Ba + Bs (combination): Combined application of *B. amyloliquefaciens* and *B. subtilis*; NB, Nutrient broth (negative control); SDW, Sterile distilled water (negative control); VP, Velum Prime^®^ (500 g a.i./ha) as the positive control. All treatments were assessed at various exposure times (in hours), and their efficacy was evaluated based on the mortality rate of *M. incognita* second-stage juveniles (J2s).

The efficacy of biocontrol agents against *M. incognita* juveniles (J_2_s) was evaluated under *in vitro* laboratory conditions. The data presented in **Figure 1** and **Supplementary Table 1** indicate that cell-free culture filtrates of two bio-control agents and a combination formulation significantly increased J_2_ mortality (*P* < 0.05). A clear dose- and time-dependent trend was observed, where higher concentrations and prolonged exposure times resulted in greater nematode mortality. After 96 hours of exposure, the highest juvenile mortality (92.67%) was recorded in the combination treatment (*B. subtilis* + *B. amyloliquefaciens*) at 100% concentration, which was significantly higher than the individual isolates *B. amyloliquefaciens* (74.67%) and *B. subtilis* (72.34%). The effectiveness of both individual isolates and the combination remained evident even at lower concentrations (50% and 25%), where juvenile mortality ranged between 50% and 75% after 96 hours. These findings confirm that the combined application of bio-control agents has a synergistic effect, enhancing nematicidal activity against *M. incognita*. Notably, no juvenile mortality was observed in the control treatments (nutrient broth and distilled water), reaffirming that the observed nematicidal effects were attributed to the bio-control agents.

### Egg hatching inhibition activity of biocontrol agents

**Figure 2:**
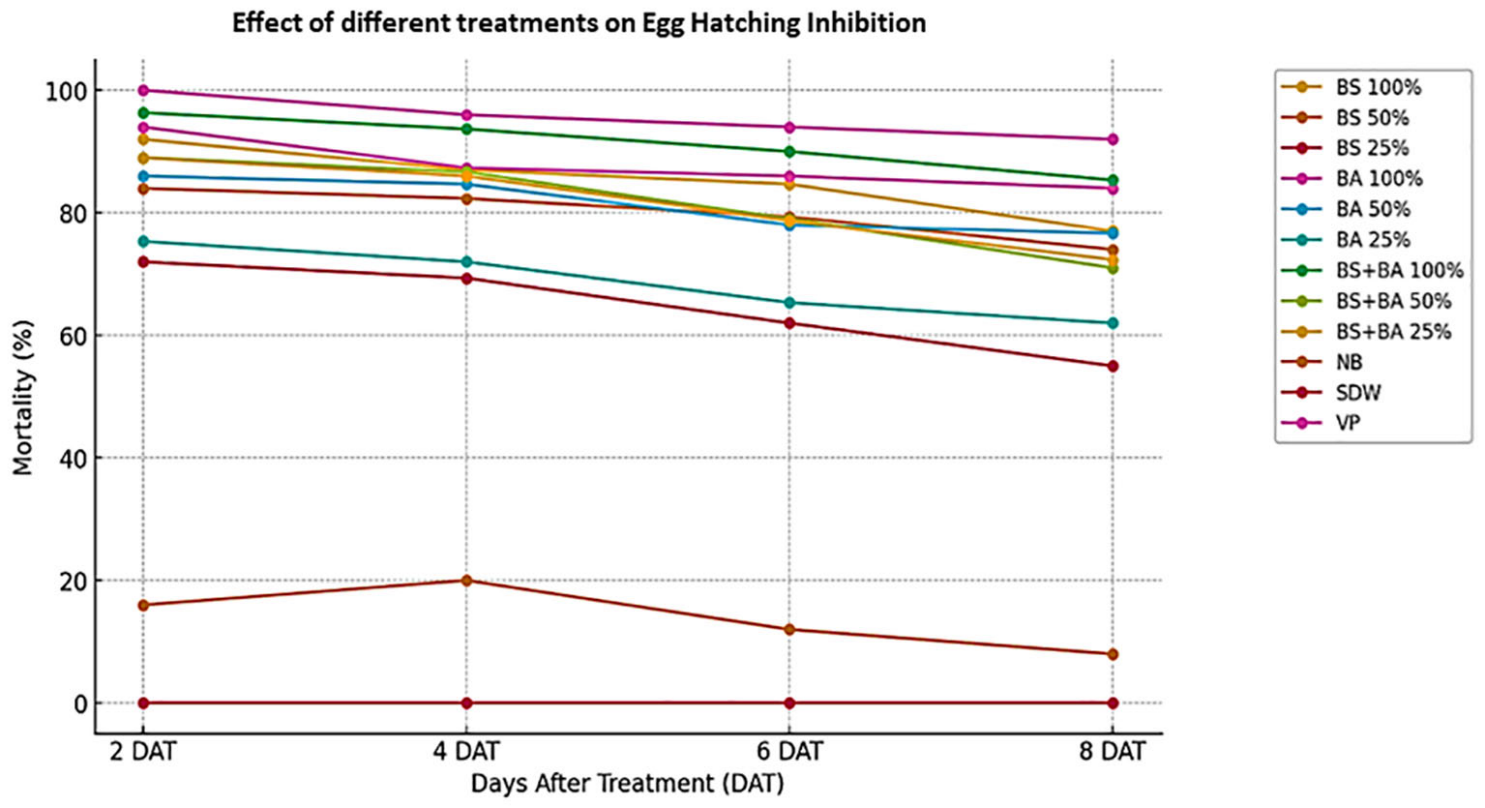
Bio-efficacy of bio-control agents on egg hatching inhibition of *Meloidogyne incognita*. Abbreviations and Treatment Details: Ba, *Bacillus amyloliquefaciens* (strain DSBA 11); Bs, *Bacillus subtilis* (strain DTBS 5); Ba + Bs (combination), Combined application of *B. amyloliquefaciens* and *B. subtilis*; NB, Nutrient broth (negative control); SDW, Sterile distilled water (negative control); VP, Velum Prime^®^ (500 g a.i./ha) as the positive control. Egg hatching inhibition was evaluated at different time intervals (days after treatment—DAT).

The efficacy of bio-control agents in inhibiting *M. incognita* egg hatching was assessed under laboratory conditions. The data presented in **Figure 2** and **Supplementary Table 2** indicate that both individual bio-control agents and their combination exhibited significant inhibition of egg hatching (*P* < 0.05), with effectiveness varying based on concentration and exposure duration. After 8 days of incubation, the highest hatching inhibition (85.34%) was observed in the combinationtreatment (*B. subtilis* + *B. amyloliquefaciens*) at 100% concentration, which was slightly higher than the inhibition rates of *B. amyloliquefaciens* (84%) and *B. subtilis* (77%) at the same concentration. A concentration-dependent trend was evident, as all treatments, including those at 50% and 25% concentrations, significantly reduced egg hatching. The inhibition effect increased with both concentration and incubation period, highlighting the potency of bio-control agents in disrupting nematode reproduction. Among all the tested treatments, the combination (*B. subtilis* + *B. amyloliquefaciens*) consistently exhibited the highest efficacy across all concentrations. In contrast, no egg hatching inhibition was observed in the distilled water control, while a minimal inhibition rate of 8% was recorded in the nutrient broth after 8 days.

### Nematicidal activity of oils and extracts

The nematicidal activity of essential oils and hexane extract from *M.* sp*icata* and *P. longum* was carried out against *M. incognita* juveniles (J_2_s), and the results are presented in **Table 2**. Results indicated that *M.* sp*icata* and *P. longum* oil exhibited significantly higher nematicidal activity compared to the hexane extracts of both *M.* sp*icata* and *P. longum* (P < 0.05). A dose and time-dependent trend was observed, where higher concentrations (500 and 1000 ppm) and prolonged exposure durations (72 and 96 hours) resulted in greater J_2_ mortality compared to lower concentrations (250 and 500 ppm) with shorter exposure times (24 and 48 hours). After 96 hours of exposure, the highest mortality was recorded in *P. longum* oil (88.67%) at 1000 ppm, followed by *M.* sp*icata* oil (85.0%). In contrast, the hexane extract of *P. longum* demonstrated relatively lower efficacy, causing 75.67% mortality and that of *M.* sp*icata* causing 72.67% mortality. The results confirm that essential oils were more effective than their respective hexane extracts, emphasizing their potential as potent natural nematicides. No juvenile mortality was observed in the negative controls, confirming that the recorded nematicidal effects were attributed solely to the botanical treatments.

**Table 2:** Bio-efficacy of different botanicals on juvenile (J2s) mortality of *Meloidogyne incognita*.

Treatments	Conc. (in ppm)	24 hr	48 hr	72 hr	96 hr
MSO	1000	73.34 ± 1.21 ^a^	78 ± 1 ^a^	80 ± 1.53 ^b^	85 ± 2.09 ^c^
	500	60 ± 1.53 ^d^	64 ± 1 ^c^	66.34 ± 1.21 ^d^	69 ± 0.58 ^e^
	250	44.34 ± 0.89 ^f^	48.34 ± 0.34 ^f^	51.34 ± 0.89 ^e^	53 ± 0.58 ^f^
	125	32.34 ± 0.89 ^g^	35.34 ± 0.34 ^h^	37.34 ± 0.89 ^f^	37 ± 0.58 ^i^
	62.5	16 ± 0.58 ^j^	17 ± 1 ^j^	21 ± 0.58 ^h^	21.67 ± 1.21 ^k^
MSH	1000	63 ± 1.16 ^c^	70 ± 0.58 ^b^	69.34 ± 0.89 ^d^	72.67 ± 1.21 ^d^
	500	48 ± 1.16 ^e^	52.34 ± 0.89 ^e^	52.34 ± 1.21 ^e^	55.67 ± 0.89 ^f^
	250	33.34 ± 0.89 ^g^	36.34 ± 1.46 ^gh^	39.67 ± 1.46 ^f^	43 ± 0.58 ^h^
	125	19 ± 0.58 ^i^	24 ± 0.58 ^i^	27 ± 0.58 ^g^	31 ± 1.16 ^j^
	62.5	7 ± 0.58 ^l^	13.34 ± 1.21 ^k^	14.34 ± 1.46 i	15.34 ± 0.89 ^l^
PLH	1000	43.34 ± 0.89 ^f^	56.67 ± 1.21 ^d^	67.34 ± 0.89 ^d^	75.67 ± 1.86 ^d^
	500	29 ± 1.16 ^h^	38.67 ± 1.21 ^g^	50.67 ± 1.21 ^e^	56.34 ± 1.46 ^f^
	250	16.34 ± 1.46 ^ij^	23.34 ± 0.89 ^i^	37.67 ± 0.89 ^f^	48 ± 2.09 ^g^
	125	11.34 ± 1.21 ^k^	19 ± 1.16 ^j^	28 ± 0.58 ^g^	36.34 ± 1.77 ^i^
	62.5	2.34 ± 0.34 ^mn^	6.67 ± 0.34 ^l^	10.34 ± 0.89 ^j^	16 ± 0.58 ^l^
PLO	1000	42.34 ± 0.89 ^f^	53 ± 1.16 ^e^	73.34 ± 0.89 ^c^	88.67± 1.77 ^b^
	500	29 ± 0.58 ^h^	37.67 ± 0.89 ^gh^	52 ± 2.09 ^e^	72.67± 2.03 ^d^
	250	15 ± 1.53 ^j^	24 ± 1.53 ^i^	28.34 ± 2.34 ^g^	45.67 ± 1.21 ^gh^
	125	7.34 ± 1.21 ^l^	13 ± 1.53 ^k^	14.34 ± 2.73 ^i^	36.34 ± 1.46 ^i^
	62.5	3 ± 0.58 ^m^	3.34 ± 0.34 ^m^	8 ± 0.58 ^j^	15.34 ± 0.67 ^l^
T-80(0.3%)		0 ± 0 ^n^	1.34 ± 0.34 ^mn^	3.34 ± 0.34 ^k^	5 ± 0 ^m^
SDW		0 ± 0 ^n^	0 ± 0 ^n^	1.34 ± 0.34 ^k^	2.34 ± 0.34 ^m^
VP		66 ± 0.58 ^b^	80 ± 0.58 ^a^	90 ± 0.58 ^a^	98 ± 0.58 ^a^
	Fcal	565.51	673.08	440.49	475.40
	CV %	5.77	4.75	5.36	4.64
	SEm±	0.96	0.95	1.24	1.26
	CD (P=0.05) 5%	2.73	2.70	3.53	3.58

Data represent the mean values of three replicates ± standard error (SE). Means within each column followed by the same letter are not significantly different (*P* < 0.05) based on the least significant difference (LSD) test. Statistical parameters include the critical difference (CD) and coefficient of variation (CV). Abbreviations and Treatment Details: MSO, *M.* sp*icata* oil; MSH, *M.* sp*icata* hexane extract; PLO, *P. longum* oil; PLH, *P. longum* hexane extract, T-80 (0.3%) – Tween 80 (used as an emulsifier control); SDW, Sterile distilled water (negative control); VP, Velum Prime^®^ (500 g a.i./ha) as the positive control. Juvenile mortality was assessed at different time intervals (*hr*), evaluating the nematicidal potential of botanical extracts.

### Egg hatching inhibition activity of oils and extracts

The data presented in **Table 3** illustrates the effect of different essential oils and extracts on the egg hatching inhibition of *M. incognita*. Overall, botanicals and essential oils exhibited greater efficacy in inhibiting egg hatching compared to hexane extracts. The inhibitory effect increased with higher concentrations, demonstrating a dose-dependent response. After 8 days, the highest hatching inhibition (95%) was recorded with *M.* sp*icata* oil at 1000 ppm, followed by *P. longum* oil (88%) at the same concentration. The lowest inhibition (75.67%) was observed in *P. longum* hexane extract at 1000 ppm. All botanical treatments exhibited significant differences (*P*<0.05) and were effective across all tested concentrations (1000, 500, 250, and 125 ppm). The results indicate that egg hatching inhibition is directly proportional to both concentration and exposure duration. In contrast, no hatching inhibition was recorded in control treatments with Tween 80 (3%) and distilled water, further confirming the nematicidal potential of the tested botanicals.

**Table 3:** Bio-efficacy of different botanicals on egg hatching inhibition of *Meloidogyne incognita*.

Treatment	Conc (in PPM)	2 DAT	4 DAT	6 DAT	8 DAT
MSO	1000	100 ± 6.25 ^a^	100 ± 1.53 ^a^	100 ± 4.17 ^a^	95 ± 0.58 ^a^
	500	93 ± 0.58 ^cd^	93.34 ± 0.34 ^bcd^	90.34 ± 0.67 ^cd^	85.34 ± 0.67 ^ef^
	250	71.67 ± 1.46 ^gh^	76.67 ± 1.21 ^g^	72.34 ± 0.89 ^fg^	71.67 ± 1.77 ^ij^
	125	66.67 ± 0.89 ^i^	71 ± 0.58 ^h^	69.34 ± 0.89 ^gh^	65.67 ± 1.21 ^l^
MSH	1000	97 ± 0.58 ^abc^	95.67 ± 0.34 ^b^	92 ± 0.58 ^bc^	91 ± 1.53 ^bc^
	500	89 ± 0.58 ^de^	86.34 ± 2.19 ^f^	83.34 ± 0.89 ^e^	75.67 ± 2.19 ^h^
	250	74.67 ± 0.89 ^fg^	76.67 ± 1.21 ^g^	71 ± 0.58 ^fg^	72.34 ± 0.89 ^i^
	125	69.34 ± 0.89 ^hi^	74.34 ± 2.03 ^g^	66.67 ± 0.89 ^h^	66.34 ± 0.89 ^kl^
PLO	1000	98.67 ± 0.34 ^ab^	96 ± 0.58 ^b^	94.34 ± 0.34 ^b^	92 ± 1.16 ^bc^
	500	96.34 ± 0.89 ^abc^	91.34 ± 0.89 ^de^	90 ± 0.58 ^cd^	88 ± 0.58 ^de^
	250	89 ± 0.58 ^de^	86.34 ± 0.89 ^f^	84.67 ± 0.34 ^e^	83 ± 0.58 ^fg^
	125	79 ± 0.58 ^f^	76 ± 0.58 ^g^	73 ± 0.58 ^f^	71 ± 0.58 ^ij^
PLH	1000	97 ± 0.58 ^abc^	94.34 ± 0.34 ^bc^	92 ± 0.58 ^bc^	89.67 ± 0.34 ^cd^
	500	95 ± 0.58 ^bc^	92 ± 0.58 ^cd^	88 ± 0.58 ^d^	86 ± 0.58 ^e^
	250	87.67 ± 0.34 ^e^	88.67 ± 0.89 ^ef^	83.34 ± 0.89 ^e^	80.67 ± 0.34 ^g^
	125	78.34 ± 1.21 ^f^	74 ± 0.58 ^g^	70 ± 0.58 ^fg^	69 ± 0.58 ^jk^
T-80(0.3%)		10 ± 0.58 ^j^	13.34 ± 0.89 ^i^	6.34 ± 0.34 ^i^	4 ± 0.58 ^m^
SDW		0 ± 0 ^k^	0 ± 0 ^j^	0 ± 0 ^j^	0 ± 0 ^n^
VP		100 ± 0	96 ± 0.58	100 ± 4.17 ^a^	95 ± 0.58 ^a^
	F cal	310.02	697.61	570.49	753.28
	CV	3.53	2.25	2.64	2.32
	SEm±	1.60	1.01	1.14	0.97
	CD (P=0.05)	4.58	2.90	3.26	2.78

Data represent the mean of three replicates ± SE. Means within each column followed by the same letter are not significantly different (P < 0.05). CD, Critical difference; CV, Coefficient of variation; DAT, Days after treatment; SE, Standard error. Treatment details: MSO, *M.* sp*icata* oil; MSH, *M.* sp*icata* hexane extract; PLO, *P. longum* oil; PLH, *P. longum* hexane extract; T-80 (0.3%), Tween 80 (emulsifier control); SDW, Sterile distilled water (negative control); VP, Velum Prime^®^ (500 g a.i./ha) as the positive control. Data are expressed as mean ± SE, and treatments were compared for significant differences at P < 0.05. Data are presented as mean ± SE, with significant differences determined at P < 0.05. F cal (Calculated F-value), SEm± (Standard Error of Mean), CV% (Coefficient of Variation), CD (P=0.05) (Critical Difference at 5% level).

### *In-vitro* comparative toxicity of botanicals on egg hatching and juvenile mortality of *M. incognita*

**Table 4** summarizes the *in vitro* toxicity (LC_50_) of various botanicals, including *M.* sp*icata* oil, *M.* sp*icata* hexane extract, *P. longum* oil, *P. longum* hexane extract, and Velum Prime, against *M. incognita*. The toxicity pattern observed after 96 hours follows the order: *M.* sp*icata* hexane extract < *P. longum* hexane extract < *P. longum* oil < *M.* sp*icata* oil. A strong correlation was observed between juvenile mortality and both exposure duration and concentration, with a maximum mortality rate of 80% recorded at higher concentrations (1000 and 500 ppm). The LC_50_ values decreased with increasing concentrations, demonstrating the efficacy of the tested oils and extracts. After 96 hours, the recorded LC_50_ values were 214.63 ppm (*M.* sp*icata* oil), 231.15 ppm (*P. longum* oil), 306.11 ppm (*P. longum* hexane extract), and 348.11 ppm (*M.* sp*icata* hexane extract), indicating that juvenile mortality increased as LC_50_ decreased.

**Table 4:** Comparative toxicity (LC 50) of different botanicals against *Meloidogyne incognita* in comparison with velum prime (VP) as a positive control.

Test samples	Exposure period		Heterogeneity			*LC 50			Fiducial Limit (ppm)			
	EHI (DAT)	JH (hours)	EHIX^2^	JH X^2^	EHI Df	JH Df	EHI ppm	JH ppm	EHI Min	JH Min.	EHI Max.	JH Max.
PLH	2	24	0.24	1.77	2	3	31.26	1,299.20	15.93	898.73	61.35	1,878.13
	4	48	2.02	2.26	2	3	25.02	806.36	11.49	563.94	54.46	1,153.00
	6	72	0.56	2.29	2	3	32.59	458.24	15.07	326.28	70.51	643.57
	8	96	0.47	2.89	2	3	30.00	306.11	12.92	216.75	69.67	432.32
PLO	2	24	0.15	0.19	2	3	40.92	1,329.53	22.99	927.03	72.84	1,906.79
	4	48	0.12	1.41	2	3	28.79	825.01	13.82	596.63	59.97	1,140.82
	6	72	0.33	1.40	2	3	29.97	476.25	14.12	365.41	63.64	620.71
	8	96	0.41	2.84	2	3	29.42	231.15	13.21	179.64	65.54	297.44
MSO	2	24	5.12	0.51	2	3	81.43	323.92	51.31	231.71	129.22	452.82
	4	48	2.73	0.70	2	3	63.23	273.91	37.66	198.90	106.15	377.22
	6	72	4.98	0.18	2	3	72.56	237.84	43.61	171.83	120.73	329.20
	8	96	2.12	0.29	2	3	70.17	214.63	39.63	159.01	124.24	289.70
MSH	2	24	2.47	0.76	2	3	65.82	549.82	38.40	401.01	112.81	753.85
	4	48	2.97	0.25	2	3	39.75	435.51	19.68	313.89	80.29	604.25
	6	72	1.58	0.41	2	3	56.94	416.27	28.78	294.09	112.67	589.21
	8	96	3.15	0.79	2	3	51.52	348.11	23.65	248.80	112.22	487.06
Velum Prime	2	24	0.57	0.38	2	3	0.60	0.04	0.10	0.01	3.51	0.20
	4	48	0.19	0.49	2	3	0.42	0.09	0.07	0.02	2.68	0.39
	6	72	0.13	0.16	2	3	0.15	0.004	0.02	0.00	1.27	0.03
	8	96	0.15	0.21	2	3	0.00	0.00	0.00	0.00	0.01	0.002

LC50 represents the lethal concentration required to achieve 50% egg hatching inhibition or 50% juvenile (J2) mortality. Data are expressed as the mean of three replicates ± SE. Means within a column followed by the same letter are not significantly different (P < 0.05). DAT, Days after treatment; CD, Critical difference; CV, Coefficient of variation; hr, Hours; SE, Standard error. Treatment details: MSO, *M.* sp*icata* oil; MSH, *M.* sp*icata* hexane extract; PLO, *P. longum* oil; PLH, *P. longum* hexane extract; VP, Velum Prime^®^ (500 g a.i./ha) as the positive control.

EHI, Egg hatching inhibition; JH, Juvenile Hatching. Data are expressed as mean ± SE, and treatments were compared for significant differences at P < 0.05. Data are presented as mean ± SE, with significant differences determined at P < 0.05. F cal (Calculated F-value), SEm± (Standard Error of Mean), CV% (Coefficient of Variation), CD (P=0.05) (Critical Difference at 5% level).

Similarly, **Table 4** also presents the *in vitro* toxicity (LC_50_) of these botanicals on egg hatching inhibition after eight days. The toxicity pattern followed the order: *M.* sp*icata* oil < *M.* sp*icata* hexane extract < *P. longum* hexane extract < *P. longum* oil. Maximum hatching inhibition was recorded at higher concentrations (1000 and 500 ppm). The LC_50_ values after eight days were 29.42 ppm (*M.* sp*icata* oil), 30.00 ppm (*M.* sp*icata* hexane extract), 51.52 ppm (*P. longum* hexane extract), and 70.17 ppm (*P. longum* oil), demonstrating that a lower LC_50_ value corresponds to a higher rate of egg hatching inhibition.

### *In vivo* experiments

#### Soil nutrient analysis (N, P, K)

Soil samples were collected from all microplots before and after treatment application to analyze nitrogen (N) content using the Kjeldahl method, phosphorus (P) using the Olsen method, and potassium (K) using a flame photometer. After 90 days of treatment, a significant increase in N, P, and K content was observed in plots treated with farmyard manure (FYM), vermicompost (VC), paddy straw (PS), and a combination of *B. subtilis* (BS) and *B. amyloliquefaciens* (BA). Among the organic amendments, FYM exhibited the highest increase in nutrient content, following the trend: FYM > VC > PS. The highest recorded values were nitrogen (263.42 kg/ha), phosphorus (378.99 kg/ha), and potassium (357.62 kg/ha) in plots treated with FYM (5–10 kg/plot). In contrast, plots without organic amendments or bio-agents showed a slight decline in NPK content. A significant difference (P < 0.05) was observed in NPK levels before and after treatment application across all treatments. Notably, the untreated control exhibited a drastic reduction in NPK content, emphasizing the role of organic and biological amendments in improving soil fertility (**Table 5**).

**Table 5:** Analysis of soil nutrient and organic carbon content under different treatments.

Treatments	Components	Before Application				After Application			
		N (kg/ha)	P (mg/kg^1^)	K (%)	OC (%)	N (kg/ha)	P (mg/kg^1^)	K (%)	OC (%)
T1	FYM	126.81 ± 1.38 ^h^	330.85 ± 1.72 ^i^	144.9 ± 2.14 ^k^	0.21 ± 0.001 ^h^	263.42 ± 0 ^a^	364.94 ± 1.86 ^i^	333.48 ± 1.22 ^b^	0.96 ± 0.002 ^a^
T2	VC	151.3 ± 0.72 ^g^	331.48 ± 1.77 ^i^	153.81 ± 0.83 ^j^	0.23 ± 0.01 ^g^	251.56 ± 0.68 ^b^	378.99 ± 1.35 ^gh^	357.62 ± 0.74 ^a^	0.89 ± 0.004 ^b^
T3	PS	113.42 ± 1.06 ^i^	308.08 ± 1.26 ^k^	164.99 ± 1.77 ^h^	0.26 ± 0.015 ^f^	219.63 ± 3.43 ^c^	354.1 ± 4.55 ^j^	268.75 ± 5.09 ^c^	0.82 ± 0.002 ^c^
T4	BS TALC	89.79 ± 1.24 ^k^	326.14 ± 1.61 ^j^	192.4 ± 0.47 ^a^	0.2 ± 0.003 ^h^	166.87 ± 2.6 ^g^	336.98 ± 1.59 ^j^	194.28 ± 0.73 ^d^	0.41 ± 0.002 ^jk^
T5	BA TALC	101.57 ± 4.37 ^j^	356.76 ± 0.61 ^g^	169.64 ± 0.86 ^def^	0.16 ± 0.01 ^i^	176.32 ± 1.02 ^f^	356.83 ± 1.72 ^h^	175.87 ± 1.35 ^fg^	0.41 ± 0.002 ^k^
T6	BS LIQUID	87.65 ± 1.15 ^k^	356.76 ± 2.21 ^g^	167.21 ± 1.02 ^fgh^	0.05 ± 0.002 ^j^	189.93 ± 0.95 ^e^	358.43 ± 0.63 ^h^	172.12 ± 1.29 ^gh^	0.42 ± 0.001 ^jk^
T7	BA LIQUID	115.58 ± 1.46 ^i^	352.39 ± 1.29 ^d^	134.66 ± 1.97 ^l^	0.31 ± 0.001 ^e^	177.04 ± 1.43 ^f^	354.82 ± 1.38 ^de^	146.78 ± 0.78 ^m^	0.53 ± 0.002 ^f^
T8	BS+BA (LIQUID)	71.96 ± 1.69 ^l^	356.43 ± 0.98 ^g^	143.42 ± 1.41 ^k^	0.32 ± 0.002 ^e^	200.36 ± 0.82 ^d^	357.54 ± 1.86 ^h^	152.04 ± 1.18 ^k^	0.51 ± 0.001 ^g^
T9	MSO 1000	216.81 ± 1.82 ^e^	351.92 ± 0.97 ^h^	183.95 ± 0.41 ^b^	0.34 ± 0.007 ^d^	127.25 ± 1.2 ^l^	349.06 ± 1.25 ^fg^	177.97 ± 0.89 ^f^	0.53 ± 0.009 ^f^
T10	MSO 2000	316.2 ± 1.37 ^b^	359.55 ± 0.88 ^fg^	173.05 ± 0.3 ^c^	0.36 ± 0.001 ^d^	137.67 ± 1.11 ^k^	335.36 ± 1.21 ^ef^	168.42 ± 0.58 ^hi^	0.35 ± 0.007 ^l^
T11	MSH 1000	312.29 ± 1.21 ^b^	376.37 ± 1.19 ^d^	143.6 ± 0.98 ^k^	0.26 ± 0.002 ^f^	151.25 ± 1.11 ^ij^	328.12 ± 2.83 ^d^	140.28 ± 0.52 ^n^	0.25 ± 0.001 ^m^
T12	MSH 2000	228.12 ± 1.21 ^d^	362.68 ± 1.47 ^ef^	158.08 ± 0.77 ^i^	0.25 ± 0.014 ^fg^	134.7 ± 2.5 ^k^	322.08 ± 0.86 ^fg^	151.57 ± 0.95 ^kl^	0.22 ± 0.002 ^n^
T13	PLO 1000	322.84 ± 1.7 ^a^	395.47 ± 1.76 ^b^	194.09 ± 0.79 ^a^	0.54 ± 0.011 ^c^	152.36 ± 1.22 ^ij^	331.23 ± 0.7 ^c^	188.75 ± 1.86 ^e^	0.43 ± 0.004 ^i^
T14	PLO 2000	249.81 ± 0.61 ^c^	384.57 ± 1.74 ^c^	172.64 ± 0.97 ^cd^	0.56 ± 0.001 ^c^	134.91 ± 1.54 ^k^	332.14 ± 1.04 ^de^	165.85 ± 1 ^i^	0.43 ± 0.002 ^ij^
T15	PLH 1000	311.89 ± 0.87 ^b^	311.32 ± 1.04 ^a^	169.04 ± 0.09 ^efg^	0.55 ± 0.009 ^c^	161.37 ± 1.14 ^h^	309.26 ± 1.1 ^a^	156.5 ± 1.16 ^j^	0.46 ± 0.014 ^h^
T16	PLH 2000	252.2 ± 1.19 ^c^	315.57 ± 0.84 ^a^	156.02 ± 1.51 ^ij^	0.89 ± 0.006 ^a^	153.68 ± 1.7 ^i^	310.51 ± 2.15 ^b^	147.16 ± 0.9 ^lm^	0.69 ± 0.005 ^e^
T17	VP	154.45 ± 1.14 ^g^	373.64 ± 1.17 ^d^	166.1 ± 0.1 ^gh^	0.82 ± 0.004 ^b^	148.16 ± 0.75 ^j^	374.01 ± 1.47 ^d^	159.7 ± 0.51 ^j^	0.72 ± 0.003 ^d^
T18	UNTREATED	161.28 ± 0.96 ^f^	366.47 ± 1.91 ^e^	170.46 ± 0.59 ^cde^	0.84 ± 0.003 ^b^	101.57 ± 4.37 ^m^	309.41 ± 1.9 ^k^	130.46 ± 0.55 ^op^	0.21 ± 0.001 ^n^
	F cal	3090.37	383.74	220.33	1160.11	532.44	193.16	1738.08	2112.05
	SEm±	1.60	1.42	1.11	0.01	1.84	1.86	1.55	0.00
	CV %	1.47	0.68	1.17	3.17	1.89	0.89	1.43	1.62
	CD (P=0.05)	4.58	4.07	3.19	0.02	5.29	5.33	4.45	0.01

Treatments included FYM (Farmyard Manure), VC (Vermicompost), PS (Paddy Straw), BS Talc (*Bacillus subtilis* talc formulation), BA Talc (*B. amyloliquefaciens* talc formulation), BS Liquid (*B. subtilis* liquid formulation), BA Liquid (*B. amyloliquefaciens* liquid formulation), BS+BA (combinationof *B. subtilis* and *B. amyloliquefaciens* liquid formulation) MSO (*Mentha* sp*icata* oil at 1000 and 2000 ppm), MSH (*M.* sp*icata* hexane extract at 1000 and 2000 ppm), PLO (*Piper longum* oil at 1000 and 2000 ppm), PLH (*P. longum* hexane extract at 1000 and 2000 ppm), and VP (Velum Prime^®^ as a positive control). Data are expressed as mean values with standard errors. Each bioagent used were enriched with well-decomposed farmyard manure (FYM) prior to application.

#### Organic carbon analysis (Walkley-Black chromic acid wet oxidation method)

Soil organic carbon content was determined using the Walkley-Black chromic acid wet oxidation method. After 90 days, a notable increase in organic carbon was recorded in plots treated with FYM, VC, PS, and *Bacillus* spp. The highest increase was observed in FYM-treated plots, following the trend: FYM > VC > PS. The highest organic carbon content (0.96%) was recorded in plots treated with FYM (5–10 kg/plot). Conversely, treatments lacking organic amendments exhibited a slight decrease in organic carbon content. A significant difference was observed among all treatments (P < 0.05), as well as between pre- and post-treatment applications. In the untreated control, a drastic reduction in organic carbon content (0.22%) was noted, further supporting the efficacy of organic amendments in enhancing soil health (**Table 5**).

#### Effect of treatments on plant growth parameters

**Figure 3:**
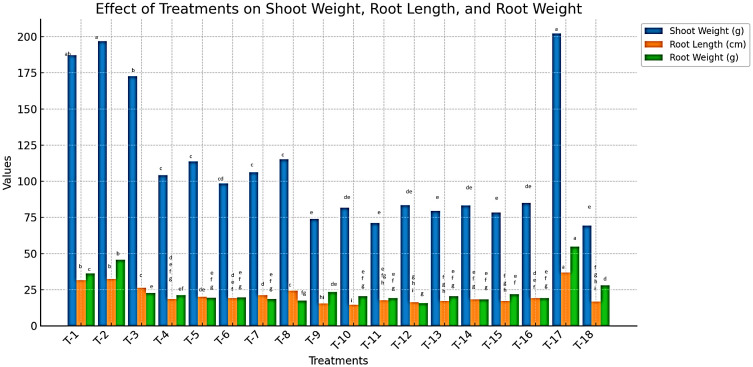
Effect of organic nutrient sources, bio-control agents, and botanicals on plant growth parameters in tomato infected by *M. incognita* under microplot.

Plant growth parameters, including shoot length, shoot weight, root length, and root weight, were significantly influenced by the application of vermicompost (VC), farmyard manure (FYM), paddy straw (PS), and the *Bacillus* spp. combination (*B. subtilis* + *B. amyloliquefaciens*) (**Supplementary Table 4**). Data presented in **Figure 3** indicate a substantial increase in plant growth across all treated plots compared to untreated controls (*P* < 0.05). After 90 days of treatment application, the maximum shoot length was recorded in vermicompost-treated plots (121.17 cm), followed by FYM (112.7 cm) and paddy straw (80.27 cm). In contrast, the lowest shoot length (ranging from 51.9 cm to 57.2 cm) was observed in plots treated with botanical extracts, including *M.* sp*icata* oil (MSO) and hexane extract (MSH), as well as *P. longum* oil (PLO) and hexane extract (PLH), at both 1000 ppm and 2000 ppm concentrations. Notably, plants treated with Velum Prime recorded the highest shoot length of 134.14 cm (**Supplementary Table 3**).

Similarly, shoot weight followed the same trend, with the highest fresh shoot weight recorded in VC-treated plants (196.83 g), followed by FYM (187.24 g) and paddy straw (172.63 g). The lowest shoot weight (71.03 g to 84.96 g) was observed in plots treated with botanical extracts at 1000 and 2000 ppm concentrations, while Velum Prime-treated plants exhibited the highest fresh shoot weight (202.13 g).

Root growth also showed significant improvement in response to organic amendments. The maximum root length was observed in VC-treated plants (32.5 cm), followed by FYM (31.47 cm) and paddy straw (26.2 cm). The shortest root lengths (14.47 cm to 19.24 cm) were recorded in botanical extract treatments, while Velum Prime-treated plants had the highest root length (36.9 cm).

Likewise, root weight was highest in VC-treated plants (45.76 g), followed by FYM (36.2 g) and paddy straw (22.73 g). The lowest root weight (15.73 g to 21.93 g) was recorded in botanical extract treatments, whereas Velum Prime-treated plants exhibited the highest fresh root weight (54.83 g).

Overall, the results revealed that organic amendments significantly improved plant growth, following the trend: VC > FYM > PS. A direct correlation was observed between shoot length, shoot weight, root length, and root weight with the applied treatments. Shoot length measurements were taken at 30, 60, and 90 days, confirming a progressive increase over time. These findings emphasize the role of organic amendments in promoting plant growth and resilience against *M. incognita* infestation.

#### Effect of treatments on nematode reproduction parameters

**Figure 4:**
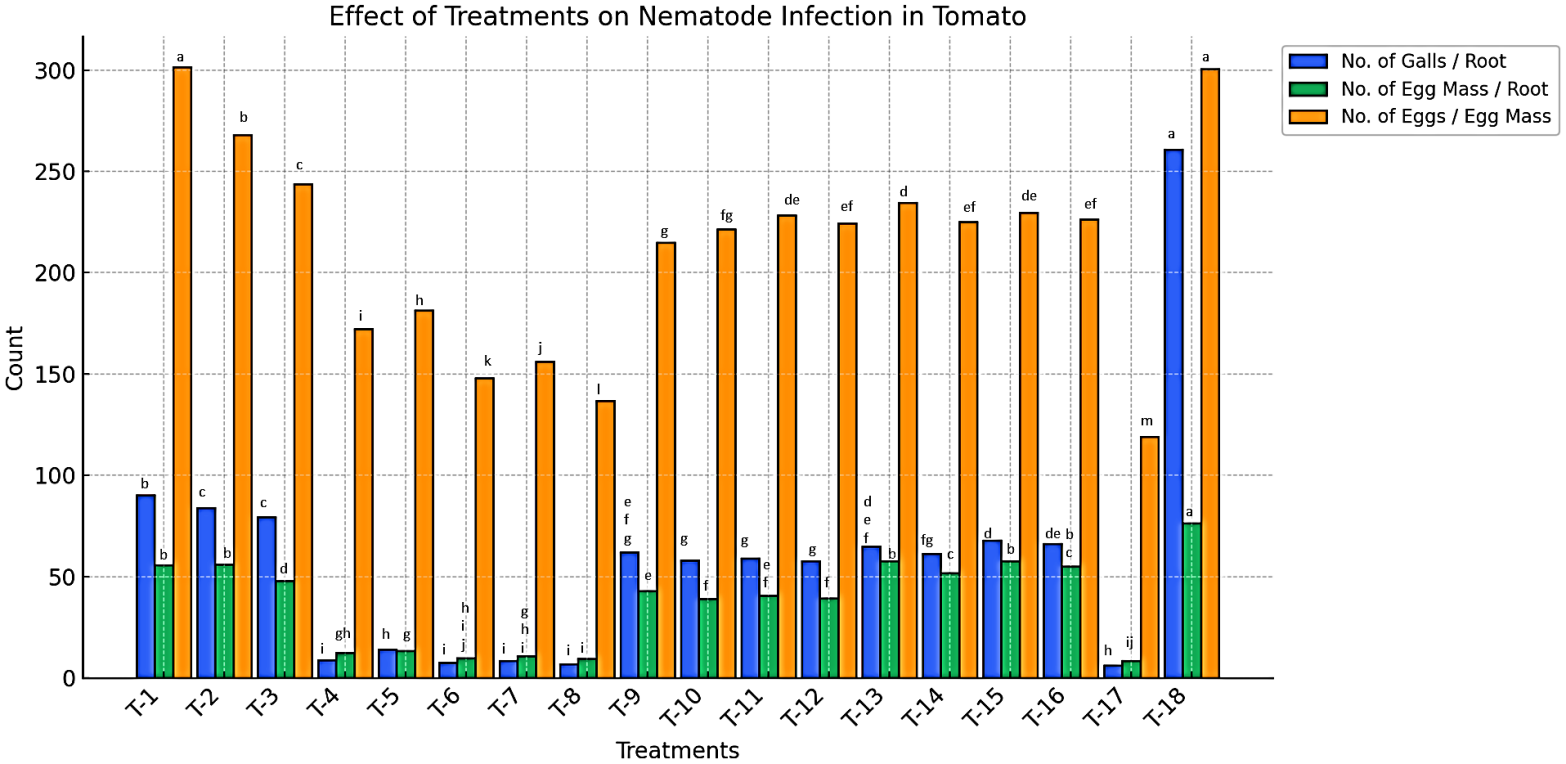
Effect of organic nutrient sources, bio-control agents, and botanicals on nematode infection in tomato infected by *M. incognita* under microplot.

Data presented in **Figure 4** indicate a significant reduction in nematode gall formation in plots treated with bio-control agents in various formulations (*P* < 0.05). The lowest number of root galls (6 galls/root) was recorded in plots treated with the *B. amyloliquefaciens* + *B. subtilis* (BA+BS) combination. This was followed by plots treated with *B. subtilis* (BS) and *B. amyloliquefaciens* (BA) in liquid formulations applied via soil drenching, which exhibited 6.67 to 8.67 galls/root (**Supplementary Table 5**). In contrast, the highest number of galls was recorded in plots treated with organic amendments such as farmyard manure (FYM), vermicompost (VC), and paddy straw (PS). Similarly, a reduction in egg mass formation was observed, with the lowest number of egg masses (6.67 egg masses/root) recorded in plots treated with the BA+BS combination. This was followed by plots treated with BS and BA (talc and liquid formulations), applied via soil drenching, which recorded 9.33 to 13.33 egg masses/root. Conversely, the highest number of egg masses (48 to 55.67 egg masses/root) was observed in plots treated with FYM, VC, and PS applied as broadcasting amendments (**Supplementary Table 5**).

Botanical treatments also exhibited a notable reduction in nematode reproduction. Plots treated with *M.* sp*icata* oil (MSO), *M.* sp*icata* hexane extract (MSH), *P. longum* oil (PLO), and *P. longum* hexane extract (PLH) at concentrations of 1000 ppm and 2000 ppm showed reduced gall formation (58 to 68 galls/root) and egg mass production (39 to 57.67 egg masses/root). Egg production per egg mass followed a similar trend. The lowest number of eggs per egg mass (136.67 eggs/egg mass) was recorded in the BA+BS combination-treated plots, followed by plots treated with BS and BA (talc and liquid formulations), which recorded 148 to 172.33 eggs per egg mass. In contrast, the highest number of eggs per egg mass (243.67 to 301.67 eggs/egg mass) was observed in plots treated with FYM, VC, and PS. Botanical treatments also effectively reduced egg production, with plots treated with MSO, MSH, PLO, and PLH (1000 and 2000 ppm) recording 215 to 234.67 eggs per egg mass. All treatments significantly reduced nematode reproduction parameters compared to untreated controls (*P* < 0.05). The reduction in the number of galls, egg masses, and eggs per egg mass was directly proportional to the type, formulation, and concentration of the treatment applied. Higher concentrations resulted in a greater reduction in nematode infection levels, highlighting the potential of bio-control agents, botanicals, and organic amendments as effective alternatives to synthetic nematicides.

## Discussion

This study demonstrates the potential of essential oils, plant extracts, biocontrol agents, and organic amendments as effective alternatives to chemical nematicides for managing *Meloidogyne incognita* in tomato. An *in vitro* evaluation of various botanicals—*Mentha* sp*icata* oil, *M.* sp*icata* hexane extract, *Piper longum* oil, and *P. longum* hexane extract—revealed that nematode mortality and egg hatching inhibition were both concentration- and time-dependent. Among the treatments, the essential oils of *P. longum* and *M.* sp*icata* showed significantly higher nematicidal activity than their respective hexane extracts, indicating their superior bioefficacy. Our findings, consistent with Taimoor and Shahina (2018), confirm that nematode mortality and egg hatching inhibition are both dose- and time-dependent. Joyamati et al. (2013) reported that petroleum ether extracts of *Melia azedarach* were highly effective in inhibiting nematode egg hatching at 1000 ppm, aligning with our findings that nematode mortality and egg hatching inhibition are dose- and time-dependent. The nematicidal activity observed in our study can be attributed to bioactive compounds such as carvone in *M.* sp*icata* oil and piperine in *P. longum* oil. Essential oils, comprising both polar and non-polar compounds, are known to disrupt nematode neuromuscular activity by inhibiting acetylcholinesterase (Rhode, 1960). Similar results were reported by Kimbaris et al. (2017), who identified piperitenone epoxide as the most potent compound in *M.* sp*icata* oil against *M. javanica*. Additionally, *M. longifolia* oil and its major component, piperitenone oxide, showed significant activity against *M. incognita* (Gowda et al., 2023). The microplot experiment revealed significant differences among treatments, suggesting the effectiveness of botanicals in enhancing plant growth and suppressing nematode populations. However, prolonged exposure to higher concentrations of *M.* sp*icata* and *P. longum* oils and their hexane extracts adversely affected tomato growth. Treated plots showed marked reductions in shoot and root development compared to the control, indicating potential phytotoxicity at elevated concentrations. These findings highlight the importance of optimizing application rates to achieve effective nematode control while minimizing negative impacts on plant health. Essential oils, particularly carvone from *M.* sp*icata* and piperine from *P. longum*, were most effective in inducing juvenile mortality and inhibiting egg hatching of *M. incognita*, likely due to their neurotoxic and enzymatic inhibition properties (Caboni et al., 2013; Morcia et al., 2016; Rajasekharan et al., 2020; Kumar et al., 2023; Mondal et al., 2024).

In the present study, *B. amyloliquefaciens* (BA), *B. subtilis* (BS), and their combination (BS + BA) were evaluated under laboratory conditions for their effects on *M. incognita* juvenile mortality and egg hatching inhibition. The enhanced nematicidal activity of the *B. amyloliquefaciens* and *B. subtilis* combination likely stems from their synergistic secretion of bioactive compounds and improved root colonization. The findings indicate a synergistic interaction between *B. amyloliquefaciens* and *B. subtilis*, enhancing their antagonistic efficacy against *M. incognita*. These results are consistent with those of Manju and Subramanian (2019), who reported increased nematode mortality through the combined action of *B. amyloliquefaciens* strain B4 and *B. subtilis* strain BG 31. Similar outcomes were observed by Alfianny et al. (2017) and Ramezani Moghaddam et al. (2014), supporting the potential of PGPR consortia in nematode biocontrol. The synergistic effect is likely due to the combined secretion of nematicidal compounds, enhanced root colonization, and improved nutrient solubilization. PGPR represent a promising biocontrol strategy for PPNs through multiple mechanisms including competition, antibiosis, mycoparasitism, lysis, and the induction of systemic resistance (Siddiqui and Mahmood, 1999; El-Nagdi and Youssef, 2004; Abdel Razek and Yaseen, 2020). Sayre and Starr (1985) further demonstrated that antagonistic PGPR produce nematicidal metabolites such as hydrogen cyanide, 2,4-diacetylphloroglucinol, gluconates, chitinases, proteases, and lipases, which inhibit nematode development and reproduction. Moreover, when used in combination with organic amendments, biocontrol agents often exhibit additive or synergistic effects, further enhancing nematode suppression (Sikora et al., 2005). Organic amendments improve soil health and foster microbial and predator populations that collectively suppress nematodes (Renco, 2013). The present study revealed that the application of FYM at 5 kg/plot resulted in the highest nitrogen content (263.42 kg/ha), while vermicompost significantly increased potassium levels (357.62 kg/ha). Additionally, the highest organic carbon content (0.96%) was observed in FYM-treated plots, suggest the beneficial impact of organic amendments on soil fertility. These findings align with those of Saha et al. (2010), who reported similar enhancements in soil nutrient status following the application of organic manures. Microplot experiments revealed a significant improvement in plant growth parameters across all organic amendment treatments. Vermicompost-treated plots recorded the highest shoot length (121.17 cm) and shoot weight (196.83 g), followed by FYM (112.7 cm, 187.24 g) and paddy straw (80.27 cm, 172.63 g). The superior performance of vermicompost may be attributed to its rich nutrient profile and its ability to enhance beneficial microbial communities that support plant growth and nematode suppression. These results are consistent with Odhiambo and Aguyoh (2019), who observed that farmyard manure reduced root galling and nematode populations in cucumber, leading to improved yields.

Despite nematode infestation, organic amendments significantly enhanced plant vigor, promoting both shoot and root development. Vermicompost-treated plants also exhibited higher concentrations of sugars, proteins, and fats compared to untreated controls. This enhancement is likely due to increased organic matter mineralization, which improves nutrient availability and releases nematicidal secondary metabolites such as ammonia, hydrogen sulfide, phenols, and organic acids (Kesba and Al-Shalaby, 2008). Additionally, organic matter supports soil biodiversity by encouraging the proliferation of nematode predators and parasitoids (Mankau, 1963).

The integration of biocontrol agents further improved nematode management. A significant reduction in nematode incidence was recorded in plots treated with the biocontrol consortium of *B. amyloliquefaciens* (BA) and *B. subtilis* (BS), which showed the lowest nematode pathogenicity. In contrast, individual applications of BA and BS were less effective, suggesting a synergistic effect in the combined treatment. Botanical treatments, *M.* sp*icata* oil (MSO) and *P. longum* hexane extract (PLH) also reduced gall formation, though to a lesser extent than the biocontrol agents.

These findings align with previous reports that biocontrol agents suppress nematodes via synergistic interactions and by inducing systemic resistance through defense-related enzymes (Meena et al., 2012; Alfianny et al., 2017). The liquid formulation of the BA+BS consortium proved particularly effective, significantly reducing gall formation and enhancing plant defense responses. Similar synergistic effects were reported by Manju and Subramanian (2019), who found that the combination of *B. subtilis* strain BG 31 and *B. amyloliquefaciens* strain B4 improved nematode suppression. Additional studies have also demonstrated the efficacy of biocontrol consortia in nematode management (Alfianny et al., 2017; Munif et al., 2013; Majzoob et al., 2012).

The potential of integrating bioagents and botanicals with organic amendments such as FYM offers a promising avenue for enhancing nematode management through synergistic effects. Although the present study primarily evaluated individual treatments and the bioagent + FYM combination, future research should focus on exploring multi-component strategies, including their influence on host resistance mechanisms such as defense-related enzyme activity. This integrated approach may lead to more sustainable and robust nematode suppression under field conditions.

## Conclusion

This study provides comprehensive insight into the efficacy of biocontrol agents, essential oils and plant extracts and organic amendments in the management of *M. incognita*. The findings demonstrate that the combined application of *B. subtilis* and *B. amyloliquefaciens* strains exhibits promising effect by enhancing nematode control activity due to egg hatching inhibition and high juvenile mortality of *M. incognita*. The essential oils from *M.* sp*icata* and *P. longum* were also found to have potent nematode control activity than extracts. Organic amendments with farmyard manure, vermicompost, and paddy straw also improved soil fertility (NPK and organic carbon content) and promoted plant growth while reducing nematode infection levels by enhancing soil fertility. Future research should focus on elucidating the molecular mechanisms underlying the nematicidal action of these treatments and evaluating their large-scale field applicability in diverse agroecosystems and crops. These findings contribute to the growing body of knowledge supporting eco-friendly nematode management strategies, paving the way for sustainable agricultural practices that minimize reliance on synthetic pesticides while ensuring crop productivity and soil health.

## Data Availability

The original contributions presented in the study are included in the article/**Supplementary Material**. Further inquiries can be directed to the corresponding author.
