# Supplementary material for: Effect of botanicals, organic nutrient sources, and bio-control agents on root-knot nematode (Meloidogyne incognita) infecting tomato
Source: Front Plant Sci. 2025 Jul 2;16:1602326. doi: 10.3389/fpls.2025.1602326 (PMC12263558; doi:10.3389/fpls.2025.1602326)
Supplement: Supplementary file 1 [file Table1.docx]

**Supplementary material**

**Effect of Botanicals, Organic Nutrient Sources, and Bio-Control Agents on Root-Knot Nematode (*Meloidogyne incognita*) Infecting Tomato**

Vimala, G.^1^, Mansi Machal^1^, Virendra Singh Rana^2^, Najam Akhtar Shakil^2^, Gautam Chawla^1^, Rashid Pervez^1^, Ashish Kumar Singh^1*^, Mukesh Jaiman^1^ and Pankaj^1**^

^1^Division of Nematology, ICAR-Indian Agricultural Research Institute, New Delhi – 110 012.

^2^Division of Agricultural Chemicals, ICAR-Indian Agricultural Research Institute, New Delhi – 110 012.

Correspondence 1*:

Ashish Kumar Singh

[ashish.singh1@icar.gov.in](mailto:ashish.singh1@icar.gov.in)

Correspondence 2**:

Pankaj

[pankaj_nema@yahoo.com](mailto:pankaj_nema@yahoo.com)

**Table 1: Bio-efficacy of different biocontrol agents on juvenile (J2) mortality of *Meloidogyne incognita.***

Abbreviations and Treatment Details: Ba: *Bacillus amyloliquefaciens* (strain DSBA 11) Bs: *Bacillus subtilis* (strain DTBS 5) Ba + Bs (Consortium): Combined application of *B. amyloliquefaciens* and *B. subtilis* NB: Nutrient broth (negative control) SDW: Sterile distilled water (negative control)

VP: Velum Prime® (500 g a.i./ha) as the positive control. Data are presented as mean ± SE, with significant differences determined at P < 0.05. F cal (Calculated F-value), SEm± (Standard Error of Mean), CV% (Coefficient of Variation), CD (P=0.05) (Critical Difference at 5% level)

| **Treatment** | **Concentration** | **24 h** | **48 h** | **72 h** | **96 h** |
| --- | --- | --- | --- | --- | --- |
| BS | 100% | 18 ± 0.58 ^c^ | 41.34 ± 1.21 ^b^ | 72.34 ± 1.21 ^cd^ | 73 ± 0.58 ^e^ |
|  | 50% | 13.67 ± 0.34 ^ef^ | 30.34 ± 0.89 ^d^ | 65 ± 1.53 ^f^ | 70.34 ± 0.89 ^f^ |
|  | 25% | 11 ± 0.58 ^g^ | 20.34 ± 0.89 ^e^ | 50 ± 0.58 ^h^ | 57 ± 0.58 ^h^ |
| BA | 100% | 21.67 ± 1.21 ^b^ | 41.67 ± 0.34 ^b^ | 74.67 ± 0.89 ^c^ | 79.67 ± 0.34 ^c^ |
|  | 50% | 15 ± 0 ^de^ | 32 ± 1.16 ^cd^ | 69.34 ± 0.89 ^de^ | 71 ± 0.58 ^ef^ |
|  | 25% | 11.67 ± 0.34 ^g^ | 23 ± 1.53 ^e^ | 55 ± 1.53 ^g^ | 59.67 ± 0.89 ^g^ |
| BS+BA | 100% | 22 ± 0.58 ^b^ | 42.34 ± 0.89 ^b^ | 85.34 ± 2.34 ^b^ | 92.67 ± 0.89 ^b^ |
|  | 50% | 15.67 ± 0.89 ^d^ | 33.34 ± 1.86 ^c^ | 75 ± 2.09 ^c^ | 80 ± 0.58 ^c^ |
|  | 25% | 12 ± 1.16 ^fg^ | 30 ± 0.58 ^d^ | 66 ± 1 ^ef^ | 75.67 ± 1.46 ^d^ |
| NB |  | 0 ± 0 ^h^ | 1.34 ± 0.34 ^f^ | 3 ± 0.58 ^i^ | 6 ± 0.58 ^i^ |
| SDW |  | 0 ± 0 ^h^ | 0 ± 0 ^f^ | 0 ± 0 ^i^ | 0 ± 0 ^j^ |
| VP |  | 96 ± 0.58 ^a^ | 100 ± 0 ^a^ | 100 ± 0 ^a^ | 100 ± 0 ^a^ |
|  | F Cal | 1469.35 | 675.84 | 568.23 | 1832.58 |
|  | CV % | 5.73 | 5.13 | 3.67 | 1.96 |
|  | SEm± | 0.65 | 0.98 | 1.26 | 0.72 |
|  | CD (P=0.05%) | 1.90 | 2.85 | 3.68 | 2.10 |

**Table 2: Bio-efficacy of biocontrol agents on egg hatching inhibition of *Meloidogyne incognita***

Abbreviations and Treatment Details: Ba: *Bacillus amyloliquefaciens* (strain DSBA 11) Bs: *Bacillus subtilis* (strain DTBS 5) Ba + Bs (Consortium): Combined application of *B. amyloliquefaciens* and *B. subtilis* NB: Nutrient broth (negative control) SDW: Sterile distilled water (negative control)

VP: Velum Prime® (500 g a.i./ha) as the positive control. All treatments were assessed at various exposure times (in hours), and their efficacy was evaluated based on the mortality rate of *M. incognita* second-stage juveniles (J2s). Data are presented as mean ± SE, with significant differences determined at P < 0.05. F cal (Calculated F-value), SEm± (Standard Error of Mean), CV% (Coefficient of Variation), CD (P=0.05) (Critical Difference at 5% level)

| **Treatments** | **Concentration** | **2 DAT** | **4 DAT** | **6 DAT** | **8 DAT** |
| --- | --- | --- | --- | --- | --- |
| BS | 100% | 92 ± 0.58 ^d^ | 87 ± 0.58 ^c^ | 84.67 ± 0.34 ^c^ | 77 ± 0.58 ^c^ |
|  | 50% | 84 ± 0.58 ^g^ | 82.34 ± 0.89 ^e^ | 79.34 ± 0.89 ^d^ | 74 ± 0.58 ^d^ |
|  | 25% | 72 ± 0.58 ^i^ | 69.34 ± 0.34 ^g^ | 62 ± 0.58 ^f^ | 55 ± 0.58 ^g^ |
| BA | 100% | 94 ± 0.58 ^c^ | 87.34 ± 0.89 ^c^ | 86 ± 0.58 ^c^ | 84 ± 0.58 ^b^ |
|  | 50% | 86 ± 0.58 ^f^ | 84.67 ± 0.34 ^d^ | 78 ± 0.58 ^d^ | 76.67 ± 0.89 ^c^ |
|  | 25% | 75.34 ± 0.34 ^h^ | 72 ± 0.58 ^f^ | 65.34 ± 0.34 ^e^ | 62 ± 0.58 ^f^ |
| BS+BA | 100% | 96.34 ± 0.89 ^b^ | 93.67 ± 0.34 ^b^ | 90 ± 0.58 ^b^ | 85.34 ± 0.89 ^b^ |
|  | 50% | 89 ± 0.58 ^e^ | 86.67 ± 0.89 ^c^ | 79 ± 0.58 ^d^ | 71 ± 0.58 ^e^ |
|  | 25% | 89 ± 0.58 ^e^ | 86 ± 0.58 ^cd^ | 78.67 ± 0.34 ^d^ | 72.34 ± 0.89 ^de^ |
| NB |  | 16 ± 0.58 ^j^ | 20 ± 0.58 ^h^ | 12 ± 0.58 ^g^ | 8 ± 0.58 ^h^ |
| SDW |  | 0 ± 0 ^k^ | 0 ± 0 ^i^ | 0 ± 0 ^h^ | 0 ± 0 ^i^ |
| VP |  | 100 ± 0 ^a^ | 96 ± 0.58 ^a^ | 94 ± 0.58 ^a^ | 92 ± 0.58 ^a^ |
|  | F Cal | 3508.74 | 2537.35 | 3174.77 | 2071.53 |
|  | CV % | 1.27 | 1.44 | 1.38 | 1.77 |
|  | SEm± | 0.54 | 0.60 | 0.54 | 0.65 |
|  | CD (P=0.05) | 1.59 | 1.75 | 1.56 | 1.88 |

**Table 3. Effect of organic manures, botanicals, and biocontrol agents on shoot length of tomato infected by *Meloidogyne incognita* under field conditions**

Treatments FYM - Farmyard Manure, VC - Vermicompost, PS - Paddy Straw), bio-control agents (*Bacillus subtilis* and *B. amyloliquefaciens* in talc and liquid formulations, as well as their consortium, botanicals (MSO - *Mentha spicata* oil, MSH - *M. spicata* hexane extract, PLO - *Piper longum* oil, PLH - *P. longum* hexane extract at 1000 and 2000 ppm), and VP (Velum Prime® as a positive control). Shoot length measurements were taken at three-time intervals to assess the effectiveness of different treatments in promoting plant growth. Data are presented as mean ± SE, with significant differences determined at P < 0.05. F cal (Calculated F-value), SEm± (Standard Error of Mean), CV% (Coefficient of Variation), CD (P=0.05) (Critical Difference at 5% level).

| **Treatments** | **Components** | **30 days** | **60 days** | **90 days** |
| --- | --- | --- | --- | --- |
| T-1 | FYM | 12.47 ± 0.42 ^bc^ | 39.07 ± 0.52 ^b^ | 112.7 ± 3.2 ^c^ |
| T-2 | VC | 12.4 ± 0.49 ^bc^ | 46.73 ± 1.94 ^a^ | 121.17 ± 4.8 ^b^ |
| T-3 | PADDY STRAW | 12.37 ± 0.19 ^bc^ | 38.87 ± 1.68 ^b^ | 88.27 ± 1.38 ^d^ |
| T-4 | BS TALC | 10.13 ± 0.35 ^de^ | 26.77 ± 1.75 ^d^ | 60.53 ± 0.43 ^fgh^ |
| T-5 | BA TALC | 12.7 ± 0.42 ^ab^ | 32.4 ± 1.56 ^c^ | 67.13 ± 2.97 ^ef^ |
| T-6 | BS LIQUID | 10.97 ± 0.44 ^d^ | 23.7 ± 1.67 ^de^ | 62.4 ± 4.11 ^fg^ |
| T-7 | BA LIQUID | 11.2 ± 0.53 ^cd^ | 23.4 ± 1.16 ^e^ | 56.13 ± 2.38 ^ghi^ |
| T-8 | BS+BA | 8.5 ± 0.61 ^fg^ | 19.03 ± 0.26 ^fg^ | 52.57 ± 0.9 ^ij^ |
| T-9 | MSO 1000 | 9.2 ± 0.61 ^ef^ | 19.4 ± 0.44 ^fg^ | 70.53 ± 2.17 ^e^ |
| T-10 | MSO 2000 | 8.93 ± 0.88 ^ef^ | 18.47 ± 0.49 ^fg^ | 54.53 ± 1.19 ^hij^ |
| T-11 | MSH 1000 | 9.3 ± 0.44 ^ef^ | 19.3 ± 0.36 ^fg^ | 52.1 ± 1.22 ^ij^ |
| T-12 | MSH 2000 | 9.3 ± 0.49 ^ef^ | 21.03 ± 0.78 ^ef^ | 66.73 ± 3.26 ^ef^ |
| T-13 | PLO 1000 | 8.53 ± 0.34 ^fg^ | 18.77 ± 0.22 ^fg^ | 57.2 ± 0.81 ^ghi^ |
| T-14 | PLO 2000 | 8.27± 0.46 ^fgh^ | 17.83 ± 0.18 ^gh^ | 51.9 ± 0.81 ^ij^ |
| T-15 | PLH 1000 | 7.53 ± 0.2 ^ghi^ | 17.67 ± 0.12 ^gh^ | 53.37 ± 0.52 ^ij^ |
| T-16 | PLH 2000 | 7.1 ± 0.12 ^hi^ | 16.97 ± 0.12 ^gh^ | 52.46 ± 0.88 ^ij^ |
| T-17 | VP | 13.97 ± 0.12 ^a^ | 48.57 ± 1.77 ^a^ | 134.13 ± 3.03 ^a^ |
| T-18 | UNTREATED | 6.53 ± 0.15 ^i^ | 14.93 ± 0.09 ^h^ | 48.4 ± 0.42 ^j^ |
|  | F cal | 23.42 | 98.00 | 126.49 |
|  | SEm± | 0.45 | 1.08 | 2.33 |
|  | CV % | 7.77 | 7.27 | 5.75 |
|  | CD (P=0.05) | 1.28 | 3.09 | 6.68 |

**Table 4. Effect of organic nutrient sources, bio-control agents, and botanicals on plant growth parameters and nematode multiplication in tomato infected by *Meloidogyne incognita* under micro plot conditions**

This table presents the impact of various organic amendments (FYM - Farmyard Manure, VC - Vermicompost, PS - Paddy Straw), bio-control agents (*Bacillus subtilis* and *B. amyloliquefaciens* in talc and liquid formulations, as well as their consortium), and botanicals (MSO - *Mentha spicata* oil, MSH - *M. spicata* hexane extract, PLO - *Piper longum* oil, PLH - *P. longum* hexane extract at 1000 and 2000 ppm) on plant growth parameters (shoot length, root length, shoot weight, and root weight) and nematode reproduction (gall formation, egg masses per root, and eggs per egg mass). Velum Prime® (VP) was included as a positive control. Data are expressed as mean ± SE, and treatments were compared for significant differences at P < 0.05. Data are presented as mean ± SE, with significant differences determined at P < 0.05. F cal (Calculated F-value), SEm± (Standard Error of Mean), CV% (Coefficient of Variation), CD (P=0.05) (Critical Difference at 5% level)

| **Treatments** | **Components** | **Shoot weight (g)** | **Root length (Cm)** | **Root Weight (g)** |
| --- | --- | --- | --- | --- |
| T-1 | FYM | 187.23 ± 23.09 ab | 31.47 ± 2.11 b | 36.2 ± 2.33 c |
| T-2 | VC | 196.83 ± 0.96 a | 32.5 ± 1.15 b | 45.77 ± 5.87 b |
| T-3 | PADDY STRAW | 172.63 ± 1.82 b | 26.2 ± 2.37 c | 22.73 ± 1.59 e |
| T-4 | BS TALC | 104.13 ± 3.56 c | 18.53 ± 0.61 defg | 21.1 ± 0.72 ef |
| T-5 | BA TALC | 113.6 ± 4.13 c | 20.07 ± 0.59 de | 19.5 ± 0.46 efg |
| T-6 | BS LIQUID | 98.53 ± 4.89 cd | 19.27 ± 0.48 def | 19.63 ± 0.61 efg |
| T-7 | BA LIQUID | 106.27 ± 5.41 c | 21.17 ± 0.38 d | 18.53 ± 0.52 efg |
| T-8 | BS+BA | 115.27 ± 2.86 c | 24.33 ± 0.7 c | 17.4 ± 0.45 fg |
| T-9 | MSO 1000 | 73.87 ± 3.21 e | 15.47 ± 0.58 hi | 23.43 ± 1.57 de |
| T-10 | MSO 2000 | 81.63 ± 3.02 de | 14.47 ± 0.7 i | 20.53 ± 0.61 efg |
| T-11 | MSH 1000 | 71.03 ± 0.82 e | 17.77 ± 0.15 efgh | 19.23 ± 0.55 efg |
| T-12 | MSH 2000 | 83.5 ± 1.73 de | 16.23 ± 0.18 ghi | 15.73 ± 0.35 g |
| T-13 | PLO 1000 | 79.53 ± 0.58 e | 17.3 ± 0.26 fgh | 20.43 ± 0.46 efg |
| T-14 | PLO 2000 | 83.2 ± 1.73 de | 18.27 ± 0.2 efg | 18.37 ± 0.56 efg |
| T-15 | PLH 1000 | 78.37 ± 1.62 e | 17.17 ± 0.18 fgh | 21.93 ± 0.94 ef |
| T-16 | PLH 2000 | 84.97 ± 1.09 de | 19.23 ± 0.18 def | 19.2 ± 0.17 efg |
| T-17 | VP | 202.13 ± 2.05 a | 36.9 ± 0.78 a | 54.83 ± 2.56 a |
| T-18 | UNTREATED | 69.43 ± 2.58 e | 16.83 ± 0.84 fghi | 28.03 ± 1.51 d |
|  | F cal | 55.90 | 48.64 | 34.60 |
|  | SEm± | 6.10 | 0.92 | 1.79 |
|  | CV % | 9.50 | 7.51 | 12.60 |
|  | CD (P=0.05) | 17.50 | 2.65 | 5.13 |

**Table 5. Effect of organic nutrient sources, bio-control agents, and botanicals on nematode infection in tomato infected by *Meloidogyne incognita* under plot conditions**

Data shown correspond to the mean of three replicates ± SE. Means with the same alphabet letters on each column are not significantly (*P < 0.05*) different. CD: Critical difference, CV: Coefficient of variation, SE: Standard error. Treatment details: FYM-Farm yard manure; VC-Vermi compost; PS-Paddy straw; Ba- *Bacillus amyloliquefaciens* DSBA 11; Bs- *Bacillus subtilis* DTBS 5; Ba + Bs (Consortium);MSO-*Mentha spicata* oil; MSH-*Mentha spicata* hexane extract; PLO-*Piper longum* oil; PLH-*Piper longum* hexane extract;VP- Velum Prime^®^ (500 g a.i./ha) as positive control. Data are expressed as mean ± SE, and treatments were compared for significant differences at P < 0.05. Data are presented as mean ± SE, with significant differences determined at P < 0.05. F cal (Calculated F-value), SEm± (Standard Error of Mean), CV% (Coefficient of Variation), CD (P=0.05) (Critical Difference at 5% level)

| **Treatments** | **Components** | **No. of galls/**  **root** | **No. of egg mass/**  **root** | **No. of eggs/**  **egg masses** |
| --- | --- | --- | --- | --- |
| T-1 | FYM | 90.33 ± 0.88 b | 55.67 ± 2.19 ^b^ | 301.67 ± 5.55 ^a^ |
| T-2 | VC | 84 ± 1.15 c | 56 ± 1.53 ^b^ | 268 ± 1.73 ^b^ |
| T-3 | PADDY STRAW | 79.33 ± 2.03 c | 48 ± 1.73 ^d^ | 243.67 ± 1.2 ^c^ |
| T-4 | BS TALC | 9 ± 0.58 i | 12.33±0.33 ^gh^ | 172.33 ± 1.45 ^i^ |
| T-5 | BA TALC | 14 ± 1.15 h | 13.33 ± 1.45 ^g^ | 181.33 ± 1.76 ^h^ |
| T-6 | BS LIQUID | 7.67 ± 0.33 i | 9.73 ± 0.88 ^hij^ | 148 ± 2.31 ^k^ |
| T-7 | BA LIQUID | 8.67 ± 0.33 i | 10.67 ± 1.2 ^ghi^ | 156.33 ± 2.96 ^j^ |
| T-8 | BS+BA | 7 ± 0.58 i | 9.67 ± 0.33 ^i^ | 136.67 ± 2.6 ^l^ |
| T-9 | MSO 1000 | 62 ± 0.58 efg | 43 ± 1.15 ^e^ | 215 ± 1.53 ^g^ |
| T-10 | MSO 2000 | 58 ± 0.58 g | 39 ± 0.58 ^f^ | 221.33 ±1.45 ^fg^ |
| T-11 | MSH 1000 | 59 ± 1.15 g | 40.67 ±1.45 ^ef^ | 228.33±1.76 ^de^ |
| T-12 | MSH 2000 | 57.67 ± 0.88 g | 39.33 ± 1.45 ^f^ | 224.33 ± 1.76 ^ef^ |
| T-13 | PLO 1000 | 65 ± 1.73 def | 57.67 ±1.86 ^b^ | 234.67 ± 2.6 ^d^ |
| T-14 | PLO 2000 | 61.33 ± 0.33 fg | 52 ± 0.58 ^c^ | 225 ± 2.31 ^ef^ |
| T-15 | PLH 1000 | 68 ± 0.58 d | 57.67 ±0.88 ^b^ | 229.67 ± 1.2 ^de^ |
| T-16 | PLH 2000 | 66.33 ± 0.33 de | 55 ± 0.58 ^bc^ | 226.33 ± 2.73 ^ef^ |
| T-17 | VP | 6.33 ± 0.88 h | 8.67 ± 0.33 ^ij^ | 119 ± 0.58 ^m^ |
| T-18 | UNTREATED | 261 ± 5.69 a | 76.33 ± 1.2 ^a^ | 300.67 ± 2.33 ^a^ |
|  | F cal | 1267.94 | 320.66 | 490.66 |
|  | SEm± | 1.63 | 1.22 | 2.34 |
|  | CV % | 4.74 | 5.60 | 1.91 |
|  | CD (P=0.05) | 4.68 | 3.51 | 6.72 |
